# IBS-Catalyzed Regioselective Oxidation of Phenols to 1,2-Quinones with Oxone^®^

**DOI:** 10.3390/molecules17078604

**Published:** 2012-07-18

**Authors:** Muhammet Uyanik, Tatsuya Mutsuga, Kazuaki Ishihara

**Affiliations:** 1 Graduate School of Engineering, Nagoya University, Furo-cho, Chikusa, Nagoya 464-8603, Japan; 2 Japan Science and Technology Agency (JST), CREST, Nagoya University, Furo-cho, Chikusa, Nagoya 464-8603, Japan

**Keywords:** oxidation, phenol, *o*-quinone, 2-iodoxybenzenesulfonic acid (IBS), Oxone^®^

## Abstract

We have developed the first example of hypervalent iodine(V)-catalyzed regioselective oxidation of phenols to *o*-quinones. Various phenols could be oxidized to the corresponding *o*-quinones in good to excellent yields using catalytic amounts of sodium salts of 2-iodobenzenesulfonic acids (*pre*-IBSes) and stoichiometric amounts of Oxone^®^ as a co-oxidant under mild conditions. The reaction rate of IBS-catalyzed oxidation under nonaqueous conditions was further accelerated in the presence of an inorganic base such as potassium carbonate (K_2_CO_3_), a phase transfer catalyst such as tetrabutylammonium hydrogen sulfate (*n*Bu_4_NHSO_4_), and a dehydrating agent such as anhydrous sodium sulfate (Na_2_SO_4_).

## 1. Introduction

*o*-Quinones are useful synthetic intermediates for the synthesis of medicinally and biologically important compounds [1,2,3,4,5,6,7,8,9,10,11]. To date, numerous methods have been reported for the preparation of *p*-quinones by the oxidation of phenols or their derivatives [12,13,14]. For instance, the oxidation of phenols with Fremy’s radical [15], MeReO_3_ [16], dimethyldioxirane [17], or benzeneseleninic anhydride [18] mostly gives *p*-quinones, unless blocked by a substituent. However, there have been only a few studies on the direct conversion of a phenol into an *o*-quinone. In 2002, Pettus and colleagues reported the regioselective oxidation of phenols with stoichiometric amounts of 2-iodoxybenzoic acid (IBX) to the *o*-quinones [19]. After Pettus’ pioneering findings, this method was applied to the synthesis of biologically active compounds such as catecholestrogen [20], catecholamine [21], hydroxytyrosol [22], and flavonoid [23] derivatives. In 2010, Harvey and colleagues reported the regiospecific oxidation of polycyclic aromatic phenols to quinones using hypervalent iodine(III and V) reagents [24]. Accordingly, oxidation with IBX in non-aqueous DMF gives *o*-quinones, while oxidation with bis(trifluoro-acetoxy)iodobenzene in aqueous DMF gives *p*-quinones selectively.

The hypervalent organoiodine(III or V)-catalyzed oxidation reactions with co-oxidants have also been extensively investigated over the past seven years [25,26,27,28,29]. From 2007 to 2009, Yakura and colleagues reported that *p*-alkoxyphenols or *p*-arylphenols were oxidized to the corresponding *p*-quinones or *p*-quinols, respectively, in excellent yields using catalytic amounts of 4-iodophenoxyacetic acid with Oxone^®^ (2KHSO_5_•KHSO_4_•K_2_SO_4_) as a co-oxidant in aqueous acetonitrile [30,31,32]. To the best of our knowledge, however, there are no successful examples of a catalytic hypervalent iodine system for the regio-selective oxidation of phenols to *o*-quinones.

**Scheme 1:**
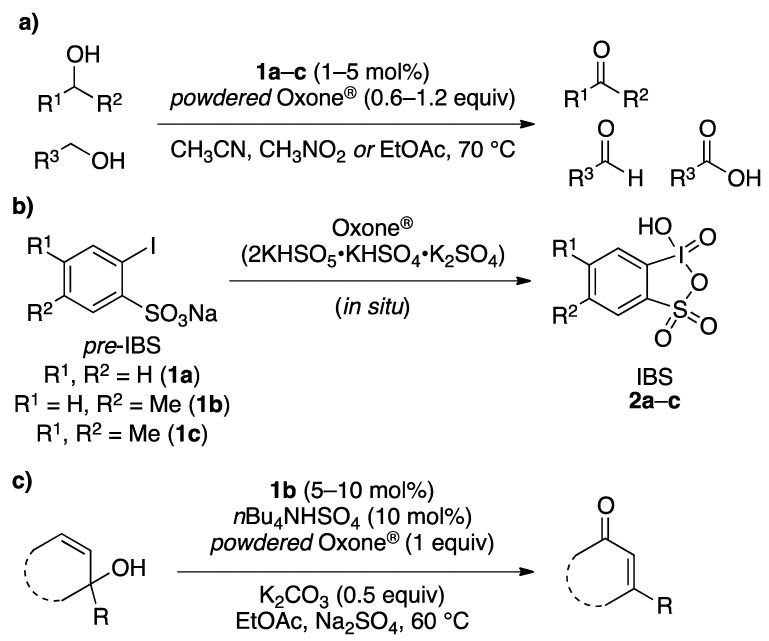
*In situ* generated IBS (**2**)-catalyzed selective oxidation of alcohols and oxidative rearrangement of tertiary allylic alcohols with *powdered* Oxone^®^ under non-aqueous conditions.

We recently reported a highly efficient and chemoselective oxidation of various alcohols to carbonyl compounds such as aldehydes, carboxylic acids, and ketones with *powdered* Oxone^®^ in the presence of catalytic amounts (1–5 mol%) of 2-iodobenzenesulfonic acids (*pre*-IBSes) or their sodium salts (**1a–c**) under nonaqueous conditions (Scheme 1a) [33,34,35,36]. 2-Iodoxybenzenesulfonic acids (IBSes) **2a–c** as iodine(V), which are generated *in situ* from **1a–c** and Oxone^®^, serve as the actual catalysts for the oxidations (Scheme 1b) [33,34,35,36]. According to previous theoretical calculations [33], the relatively ionic character of the intramolecular hypervalent iodine-OSO_2_ bond of IBS **2a** lowers the twisting barrier of the alkoxyperiodinane intermediate. In fact, **2a** shows much more catalytic activity than IBX [33]. The oxidation rate in **2a-**catalyzed oxidation under nonaqueous conditions is further accelerated by the use of *powdered *Oxone^®^ due to its increased surface area. When Oxone^®^ is used under nonaqueous conditions, Oxone^®^ wastes can be removed by simple filtration. Furthermore, we developed the oxidative rearrangement of tertiary allylic alcohols to β-disubstituted α,β-unsaturated ketones with Oxone^®^ catalyzed by *in situ-*generated 5-Me-IBS (**2b**) (Scheme 1c) [37]. The addition of inorganic bases such as K_2_CO_3_, and a phase transfer catalyst such as tetrabutylammonium hydrogen sulfate (*n*Bu_4_NHSO_4_), extended the substrate scope for oxidative rearrangement reactions. Recently, the IBS/Oxone^®^ catalytic oxidation system was applied to benzylic oxidation [38] and oxidation of fluorinated alcohols [39]. As part of our continuing interest in the IBS-catalyzed oxidation system, we report here the *in situ*-generated IBS-catalyzed regioselective oxidation of phenols to *o*-quinones with Oxone^®^.

## 2. Results and Discussion

**Scheme 2:**
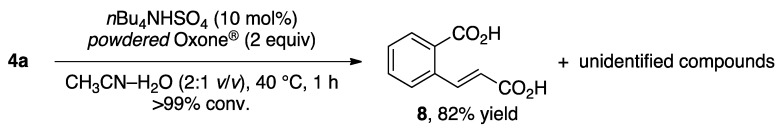
Oxidative carbon-carbon bond cleavage of **4a** to dicarboxylic acid **8** under aqueous conditions.

Initially, we investigated the reactivity and regioselectivity of the oxidation of 1-naphthol (**3a**) using conventional hypervalent catalysts under non-aqueous conditions (Table 1). A mixture of **3a**, *powdered* Oxone^®^ (2 equiv.) and *n*Bu_4_NHSO_4_ (10 mol%) as a solid-liquid phase transfer catalyst was heated in ethyl acetate at 40 °C in the presence of 5 mol% of iodobenzene or Yakura’s pre-catalyst (4-iodophenoxyacetic acid, **6**) [30,31,32] (entries 2 and 3). However only trace amounts of the desired products were detected, and more than 80% of **3a** was recovered with small amounts of unidentified side-products. The reaction was somewhat messy, and more than 80% of **3a** was recovered. Additionally, the use of *pre*-IBX (**7**) gave both 1,2-naphthoquinone (**4a**) and 1,4-naphthoquinone (**5a**) each in 5% yield, and 80% of **3a** was recovered (entry 4). In sharp contrast, and to our delight, when *pre*-IBS (**1a**) was used, **3a** was completely consumed in 11 h, and quinones **4a** and **5a** were obtained in respective yields of 64% and 5% together with highly polar compounds (entry 5). As expected from our previous works [33,37], the use of *pre*-5-Me-IBS (**1b**) or *pre*-4,5-Me_2_-IBS (**1c**) gave slightly better results, and the former gave the best results (entries 6 and 7). Interestingly, when the oxidation was conducted in aqueous acetonitrile, **5a** was obtained selectively as a major product in 51% yield (entry 8). We found that the carbon(1)-carbon(2) bond of *o*-quinone **4a** was oxidatively cleaved under identical aqueous conditions to highly polar compounds including *trans*-2-carboxycinnamic acid (**8**) [40] and other minor unidentified compounds (Scheme 2). These results indicated that non-aqueous conditions were essential for the preparation of *o*-quinones in high yields. According to our previous works, the selective oxidation of acid-sensitive alcohols could be achieved in the presence of anhydrous sodium sulfate as a dehydrating agent [33,37]. Additionally, the oxidation rate and selectivity could be further accelerated with the use of additional base to buffer the acidity of the reaction mixture [37]. Based on these previous findings, the reaction of **3a** was carried out in the presence of 1 equiv. of potassium carbonate and anhydrous sodium sulfate under the modified conditions in entry 6. Thus, **4a** was obtained in 78% yield after 1 h, when Oxone^®^ and K_2_CO_3_ were sufficiently premixed in the presence of anhydrous Na_2_SO_4_ in ethyl acetate at room temperature for 24 h before the addition of **2b**, **3a**, and *n*Bu_4_NHSO_4_ (entry 9). Notably, the use of *n*Bu_4_NHSO_4_ was essential for the present oxidation, since almost no reaction occurred in its absence (entry 10).

**Table 1:** Hypervalent iodine-catalyzed oxidation of 1-naphthol **3a**. 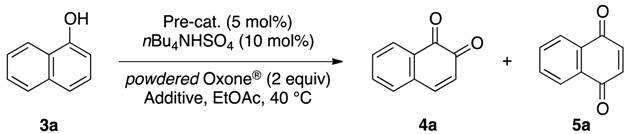

Entry	Pre-cat.	Additive (equiv.)	Time (h)	4a, Yield (%) ^a^	5a, Yield (%) ^a^
1	–	–	24	trace ^b^	trace ^b^
2	PhI	–	24	trace ^b^	trace ^b^
3	**6** ^f^	–	24	trace ^b^	trace ^b^
4	**7** ^g^	–	24	5 ^b^	5 ^b^
5	**1a**	–	11	64	5
6	**1b**	–	8	69	6
7	**1c**	–	9	67	6
8 ^c^	**1b**	–	3.5	trace ^b^	51
9 ^d^	**1b**	K_2_CO_3_ (1)	1	78	6
10 ^e^	**1b**	K_2_CO_3_ (1)	24	trace ^b^	trace ^b^

^a^ Isolated yield; ^b^^ 1^H-NMR analysis; ^c^ The reaction was performed in CH_3_CN-H_2_O (2:1, v/v) instead of EtOAc; ^d^ After a mixture of Oxone^®^ and K_2_CO_3_ in ethyl acetate was vigorously stirred in the presence of Na_2_SO_4_ for 24 h at room temperature, **1a**, **3a** and *n*Bu_4_NHSO_4_ were added; ^e^ In the absence of *n*Bu_4_NHSO_4_; ^f^** 6**: 4-Iodophenoxyacetic acid; ^g^**7**: 2-Iodobenzoic acid.

**Scheme 3:**
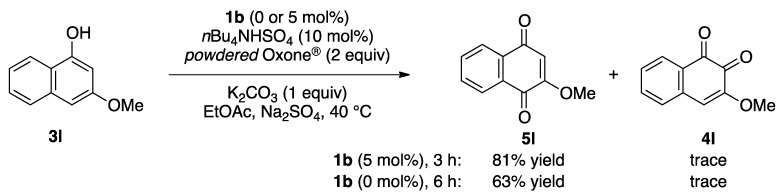
Oxidation of 3-methoxy-1-naphthol **3i**.

To explore the generality of the *in situ*-generated 5-Me-IBS-catalyzed oxidation of phenols with Oxone^®^, various naphthols, phenanthrols, and phenols **3b–l** were examined as substrates under the optimized conditions: *powdered* Oxone^®^ (2 equiv.) and potassium carbonate (1 equiv.) in ethyl acetate were vigorously stirred at room temperature for 24 h in the presence of anhydrous sodium sulfate, and then **1b** (5 mol%), **3a** and *n*Bu_4_NHSO_4_ (10 mol%) were added and the resulting mixture was heated to 40 °C (Table 2). As expected, **4a** was obtained in slightly better yield by the oxidation of 2-naphthol **3b** than by the oxidation of **3a** (Table 2, entry 1 *versus *Table 1, entry 9). 4-Bromo- or chloro-substituted 1-naphthols **3c** and **3d** gave the corresponding *o*-quinones in high yields (entries 2 and 3). Notably, the desired 1,2-quinones were obtained as a major product under our catalytic conditions even with the oxidation of 4-methoxy-1-naphthol (**3e**) and 4-methoxyphenol (**3j**) (entries 4 and 9). Accordingly, the previous iodine(III)-mediated oxidation of *para*-alkoxy phenols gave 1,4-quinones exclusively [30,31,32]. Additionally, the oxidation of phenanthrols **3g** and **3h** gave the desired 1,2-quinones in excellent yields (entries 6 and 7). These polycyclic aromatic quinones were obtained in only moderate yields by stoichiometric oxidations with IBX [24]. The oxidation of 2,4-di-*tert-*butylphenol (**3k**) gave desired *o*-quinone **4k** in 73% yield after 24 h (entry 10). In contrast, the oxidation of 3-methoxy-1-naphthol (**3l**) gave 1,4-quinone **5l** rather than 1,2-quinone **4l** as a major product (Scheme 3). Additionally, the oxidation of **3l** with Oxone^®^ even in the absence of **1b** also gave **5l** selectively, but in lower yield (63%) after 6 h.

**Table 2:** 5-Me-IBS-catalyzed oxidation of naphthols, phenanthrols and phenols **3** ^a^. 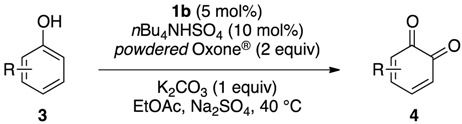

Entry	3	4	Time (h)	Yield (%) ^b^
1	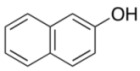	**4a**	4	84
	**3b**			
	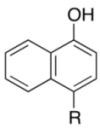	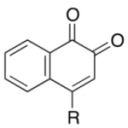		
2	**3c** (R = Cl)	**4c**	5	80
3	**3d** (R = Br)	**4d**	3	75
4	**3e** (R = OMe)	**4e**	2	50 ^c^
5	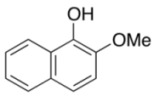	**4a**	2	72
	**3f**			
6	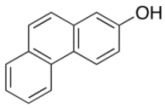	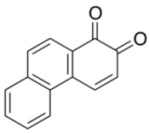	2	90
	**3g**	**4g**		
7	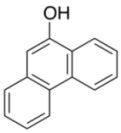	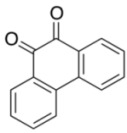	2	97
	**3h**	**4h**		
8	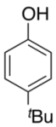	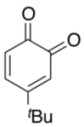	5	63
	**3i**	**4i**		
9	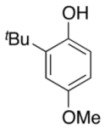	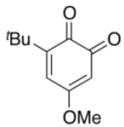	5	66 ^d^
	**3j**	**4j**		
10	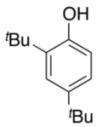	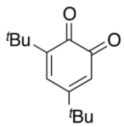	24	73
	**3k**	**4k**		

^a^ Reaction conditions: **3** (1 mmol), *powdered* Oxone^®^ (2 mmol), K_2_CO_3_ (1 mmol), **1b** (0.05 mmol), *n*Bu_4_NHSO_4_ (0.1 mmol), Na_2_SO_4_ (1 g), EtOAc (10 mL), 40 °C. Oxone^®^ and K_2_CO_3_ were pre-treated in EtOAc for 24 h at room temperature in the presence of anhydrous Na_2_SO_4_; ^b^ Isolated yield; ^c^ 1,4-Naphthoquinone (**5a**) was obtained in 15% yield; ^d^ 2-*tert*-Butyl-1,4-quinone **5j** obtained in 16% yield.

**Scheme 4:**
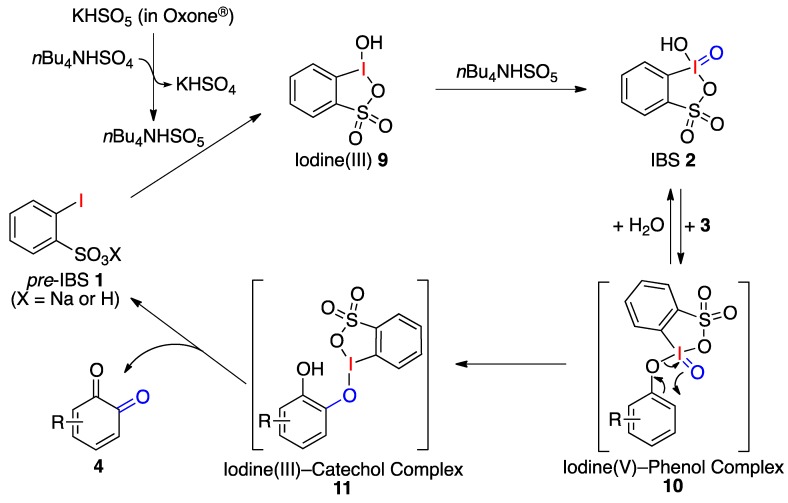
Possible mechanism for the IBS-catalyzed oxidation of phenols.

Based on previous studies [24,33,34,35,36,37], a proposed reaction mechanism is depicted in Scheme 4. *In situ*-generated IBS (**2**) reversibly combines with **3** to give IBS-phenol complex **10**, which serves to transfer oxygen from an iodoxy group (I^V^ = O) to the *ortho*-site of the phenol through concerted intramolecular [2,3]-rearrangement. During this process, the iodine(V) atom is concurrently reduced to the iodine(III)-catechol complex **11**, which gives *o*-quinones **4** and *pre*-IBS **1**. The catalytic cycle of IBS **2** can be accomplished by the regeneration of **2** through the successive oxidations of **1** and **9** with tetrabutylammonium peroxymonosulfate, *n*Bu_4_NHSO_5_, which can be generated *in situ* from KHSO_5_ and *n*Bu_4_NHSO_4_.

**Scheme 5:**
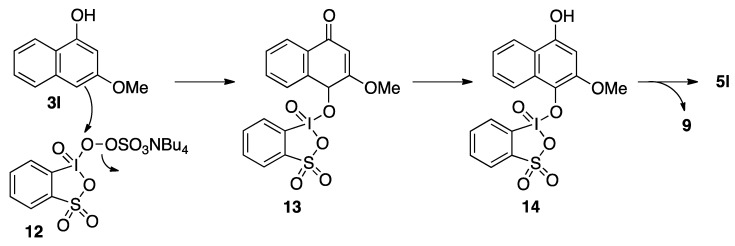
Possible mechanism for the *para*-selective oxidation of **3l**.

While, the reason for the *para*-selective oxidation of **3l** is not yet clear, a plausible mechanism is depicted in Scheme 5. The peroxo-IBS complex **12** might be generated reversibly *in situ* from IBS and ammonium Oxone^®^. Electrophilic aromatic oxidation at the highly nucleophilic carbon(4) position of **3l** with **12** gives **13**, which easily tautomerizes to IBS-hydroquinone complex **14**. Finally, the oxidation of hydroquinone gives 1,4-quinone **5l** and iodine(III) **9**. Notably, **5l** was also obtained by the oxidation of **3l** with only Oxone^®^ (Scheme 3) [41]. The reactivity of Oxone^®^ should be accelerated by complexation with IBS [42]. Thus, the oxidation was faster and the chemical yield of **5l** was higher in the presence of IBS (Scheme 3).

## 3. Experimental

### 3.1. General

Infrared (IR) spectra were recorded on a Jasco FT/IR 460 plus spectrometer. ^1^H-NMR spectra (400 MHz) and ^13^C-NMR spectra (100 MHz) were measured on a Jeol ECS-400 spectrometer at ambient temperature. Data were recorded as follows: chemical shift in ppm from internal tetramethylsilane on the δ scale, multiplicity (s = singlet; d = doublet; t = triplet; q = quartet; m = multiplet), coupling constant (Hz), integration, and assignment. Chemical shifts were recorded in ppm from the resonance of the solvent used as the internal standard (deuterochloroform at 77.0 ppm). For thin-layer chromatography (TLC) analysis throughout this work, Merck precoated TLC plates (silica gel 60 GF_254_ 0.25 mm) were used. The products were purified by column chromatography on silica gel (E. Merck Art. 9385). High-resolution mass spectral analysis (HRMS) and elemental analysis were performed at the Chemical Instrument Center, Nagoya University. Pre-catalysts **1a**–**c** were prepared according to known procedures [33]. Additionally, **1a** and **1b** (as potassium salts) are also commercially available from Junsei Chemical Japan, TCI and Sigma-Aldrich. Starting materials **3d** [43], **3f** [44], **3g** [24], and **3l** [45] were prepared according to known procedures. In experiments that required solvents, ethyl acetate, acetonitrile, and nitromethane were purchased from Wako Pure Chemical Industries, Ltd. in “anhydrous” form and used without any purification. Other simple chemicals were analytical-grade and obtained commercially.

### 3.2. General Procedure for the Oxidation Phenol to Quinone

A mixture of *powdered* Oxone^®^ (1.2 g, 2.0 mmol), potassium carbonate (0.14 g, 1.0 mmol) and anhydrous sodium sulfate (1.0 g, dried by a heat-gun under *vacuum *before use), in ethyl acetate (4.0 mL) was vigorously stirred at room temperature for 24 h. To the resulting mixture were added **3** (1.0 mmol), *n*Bu_4_NHSO_4_ (34 mg, 0.10 mmol), **1b** (17 mg, 0.050 mmol), and EtOAc (6.0 mL), and the resulting mixture was stirred vigorously at 40 °C. The reaction was monitored by TLC analysis. After the reaction was completed, the reaction mixture was cooled to room temperature and the solids were filtered-off and washed with EtOAc. The filtrate was washed with water, and the aqueous layers were extracted with EtOAc. The combined organic layers were washed by water and brine, and dried over anhydrous Na_2_SO_4_. The solvents were removed under *vacuo*, and the residue was purified by column chromatography on silica gel (hexane-EtOAc as eluent) to give the corresponding quinones **4** or **5**.

*1,2-Naphthoquinone *(**4a**) [46]. Brown solid; TLC, *R*_f_ = 0.21 (hexane–EtOAc = 4:1); ^1^H-NMR (CDCl_3_) δ 6.45 (d, *J* = 10 Hz, 1H), 7.25 (d, *J* = 8.2 Hz, 1H), 7.45 (d, *J* = 7.8 Hz, 1H), 7.53 (dd, *J* = 6.4, 7.8 Hz, 1H), 7.66 (ddd, *J* = 1.4, 5.9, 6.4 Hz, 1H), 8.13 (d, *J* = 7.3 Hz, 1H); ^13^C-NMR (CDCl_3_) δ 128.0, 130.0, 130.3, 131.0, 131.7, 134.9, 136.0, 145.6, 179.0, 181.0.

*1,4-Naphthoquinone* (**5a**) [47]. Yellow solid; TLC, *R*_f_ = 0.41 (hexane–EtOAc = 4:1); ^1^H-NMR (CDCl_3_) δ 6.99 (s, 1H), 7.77 (m, 2H), 8.10 (m, 2H); ^13^C-NMR (CDCl_3_) δ 126.6, 132.0, 134.1, 138.8, 185.2.

*trans-2-Carboxycinnamic acid* (**8**) [40]. Pale yellow solid; ^1^H-NMR (DMSO-*d*_6_) δ 6.43 (d, *J* = 16 Hz, 1H), 7.51 (t, *J* = 7.5 Hz, 1H), 7.60 (t, *J* = 6.8 Hz, 1H), 7.82 (d, *J* = 7.3 Hz, 1H), 7.88 (dd, *J* = 0.9, 7.8 Hz, 1H), 8.31 (d, *J* = 16 Hz, 1H), ^13^C-NMR (DMSO-*d_6_*) δ 121.4, 127.8, 129.8, 130.4, 131.1, 132.2, 134.9, 142.6, 167.5, 168.2.

*4-Chloro-1,2-naphthoquinone* (**4c**). Brown solid; TLC, *R*_f_ = 0.58 (hexane–EtOAc = 1:1); IR (KBr) 1,658, 1,582, 1,322, 1,287, 1,242, 936, 769 cm^−1^; ^1^H-NMR (CDCl_3_) δ 6.76 (s, 1H), 7.63 (t, *J *= 7.8 Hz, 1H), 7.77 (t, *J* = 7.8 Hz, 1H), 7.90 (d, *J *= 7.8 Hz, 1H), 8.17 (d, *J* = 7.8 Hz, 1H); ^13^C-NMR (CDCl_3_) δ 127.7, 128.0, 130.2, 130.6, 132.2, 132.7, 135.9, 152.8, 178.1, 178.4; HRMS (FAB+) *m/z* calcd for C_11_H_14_O_3_ (M+H) 193.0056, found 193.0054.

*4-Bromo-1,2-naphthoquinone* (**4d**) [48]. Brown solid; TLC, *R*_f_ = 0.62 (hexane–EtOAc = 1:1); ^1^H-NMR (CDCl_3_) δ 7.05 (s, 1H), 7.61 (t, *J *= 7.5 Hz, 1H), 7.77 (t, *J* = 7.3 Hz, 1H), 7.90 (d, *J *= 7.8 Hz, 1H), 8.15 (d, *J* = 7.3 Hz, 1H); ^13^C-NMR (CDCl_3_) δ 130.1, 130.6, 130.9, 132.1, 133.6, 136.0, 145.9, 178.2.

*4-Methoxy-1,2-naphthoquinone* (**4e**) [49]. Yellow solid; TLC, *R*_f_ = 029 (hexane–EtOAc = 1:1); ^1^H-NMR (CDCl_3_) δ 4.08 (s, 3H), 5.99 (s, 1H), 7.59 (dd, *J* = 7.3, 7.8 Hz, 1H), 7.71 (t, *J* = 7.8 Hz, 1H), 7.87 (d, *J* = 8.2 Hz, 1H), 8.13 (d, *J *= 7.3 Hz, 1H); ^13^C-NMR (CDCl_3_) δ 57.0, 103.2, 124.9, 129.2, 130.4, 131.7, 132.1, 135.1, 168.8, 179.5, 179.6.

*1,2-Phenanthraquinone* (**4g**) [24]. Red solid; TLC, *R*_f_ = 0.54 (hexane–EtOAc = 1:1); ^1^H-NMR (CDCl_3_) δ 6.59 (d, *J* = 10 Hz, 2H), 7.70 (m, 2H), 7.91 (m, 1H), 7.98 (d, *J* = 8.2 Hz, 1H), 8.17 (d, *J* = 8.2 Hz, 1H), 8.31 (m, 2H); ^13^C-NMR (CDCl_3_) δ 123.6, 124.4, 127.7, 128.6, 129.4, 129.7, 129.8, 131.4, 132.0, 137.3, 139.6, 179.5, 180.8.

*9,10-Phenanthraquinone* (**4h**) [50]. Yellow solid; TLC, *R*_f_ = 0.50 (hexane–EtOAc = 1:1); ^1^H-NMR (CDCl_3_) δ 7.47 (dd, *J* = 7.3, 7.8 Hz, 2H), 7.72 (ddd, *J* = 1.4, 6.9, 7.3 Hz, 2H), 8.03 (d, *J* = 8.3 Hz, 2H), 8.20 (dd, *J* = 1.4, 6.4 Hz, 2H); ^13^C-NMR (CDCl_3_) δ 124.1, 129.7, 130.5, 131.0, 135.9, 136.2, 180.3.

*4-tert-Butyl-1,2-benzoquinone* (**4i**) [51]. Brown solid; TLC, *R*_f_ = 0.38 (hexane–EtOAc = 1:1); ^1^H-NMR (CDCl_3_) δ 1.24 (s, 9H), 6.29 (d, *J* = 2.2 Hz, 1H), 6.40 (d, *J* = 10 Hz, 1H), 7.19 (dd, *J* = 2.5, 10 Hz, 1H); ^13^C-NMR (DMSO-*d*_6_) δ 27.4, 35.3, 123.2, 129.4, 140.2 161.5, 180.0.

*3-tert-Butyl-5-methoxy-1,2-benzoquinone* (**4j**). Red solid; TLC, *R*_f_ = 0.42 (hexane–EtOAc = 1:1); IR (KBr) 1,649, 1,630, 1,589, 1,440, 1,367, 1,228, 1,007, 900, 783; ^1^H-NMR (CDCl_3_) δ 1.26 (s, 9H), 3.84 (s, 3H), 5.73 (d, *J* = 2.7 Hz, 1H), 6.62 (d, *J* = 3.2 Hz, 1H); ^13^C-NMR (CDCl_3_) δ 28.9, 35.2 56.7, 101.0, 133.0, 151.5, 170.0, 178.6, 179.9; HRMS (FAB+) *m/z* calcd for C_11_H_14_O_3_ (M+H) 195.1021, found 195.1013.

*2-tert-Butyl-1,4-benzoquinone* (**5j**) [52]. Brown solid; TLC, *R*_f_ = 0.71 (hexane–EtOAc = 4:1); ^1^H-NMR (CDCl_3_, 400 MHz) δ 1.30 (s, 9H), 6.61 (d, *J* = 1.4 Hz, 1H), 6.69 (d, *J* = 1.4 Hz, 2H); ^13^C-NMR (CDCl_3_, 100 MHz) δ 29.2, 35.3, 131.6, 135.0, 138.7, 156.1, 188.5.

*3,5-Di-tert-Butyl-1,2-benzoquinone* (**4k**) [52]. Brown solid; TLC, *R*_f_ = 0.71 (hexane–EtOAc = 1:1); ^1^H-NMR (CDCl_3_) δ 1.23 (s, 9H), 1.27 (s, 9H), 6.22 (d, *J* = 2.3 Hz, 1H), 6.93 (d, *J* = 2.3 Hz, 1H); ^13^C-NMR (CDCl_3_) δ 28.0, 29.3, 35.6, 36.1, 122.2, 133.6, 150.0, 163.4, 180.2, 181.2.

*3-Methoxy-1,4-naphthoquinone* (**5l**) [53]. Yellow solid; TLC, *R*_f_ = 0.46 (hexane–EtOAc = 1:1); ^1^H-NMR (CDCl_3_) δ 3.90 (s, 3H), 6.17 (s, 1H); ^13^C-NMR (CDCl_3_) δ 56.6, 110.0, 126.3, 126.8, 131.1, 132.1, 133.4, 134.5, 160.5, 180.2, 185.0.

## 4. Conclusions

We have demonstrated the *in situ*-generated IBS-catalyzed regioselective oxidation of phenols to *o*-quinones with Oxone^®^. The reaction rate is accelerated with the use of inorganic bases such as K_2_CO_3_, a phase transfer catalyst such as tetrabutylammonium hydrogen sulfate (*n*Bu_4_NHSO_4_), and dehydrating agent such as Na_2_SO_4_. Various phenols are oxidized to the corresponding *o*-quinones in good to excellent yields. To the best of our knowledge, this is the first example of the hypervalent iodine-catalyzed oxidation of phenols to *o*-quinones.

## References

[1] Quideau S., Pouységu L. (1999). Synthetic uses of orthoquinonemonoketals and their orthoquinols variants. A review. Org. Prep. Proced. Int..

[2] Scott J.D., Williams R.M. (2002). Chemistry and biology of the tetrahydroisoquinoline antitumor antibiotics. Chem. Rev..

[3] Magdziak D., Meek S.J., Pettus T.R.R. (2004). Cyclohexadienoneketals and quinols: Four building blocks potentially useful for enantioselective synthesis. Chem. Rev..

[4] Pouységu L., Deffieux D., Quideau S. (2010). Hypervalent iodine-mediated phenol dearomatization in natural product synthesis. Tetrahedron.

[5] Danishefsky S.J., Mazza S., McCurry P. (1974). Diels-Alder reactions of *o*-benzoquinones. J. Org. Chem..

[6] Omote Y., Tomotake A., Kashima C. (1988). Reaction of 1,2-benzoquinones with enamines. J. Chem. Soc. Perkin Trans. 1.

[7] Nair V., Maliakal D., Treesa P.M., Rath N.P., Eigendorf G.K. (2000). [4+2] Cycloaddition reactions of *o*-benzoquinones with styrenes: A facile synthesis of bicyclo[2.2.2]octenediones. Synthesis.

[8] Stavber S., Zupan M. (1992). The effect of heteroatoms on the reactions of organic molecules with cesium fluoroxysulphate. Tetrahedron.

[9] Osman F.H., Abd El-Rahman N.M., El-Samahy F.A. (1993). Reaction of phosphonium ylides with 4-triphenylmethyl-1,2-benzoquinone. Tetrahedron.

[10] Viallon L., Reinaud O., Capdevielle P., Maumy M. (1995). Synthesis of new 4-alkylamino-5-methoxy-2H-pyran-2-ones. Tetrahedron Lett..

[11] Takuwa A., Kai R., Kawasaki K.I., Nishigaichi Y., Iwamoto H. (1996). New formal [3+2] photoaddition of vinyl ethers to *o*-benzoquinones. J. Chem. Soc. Chem. Commun..

[12] Dudfield P.J., Trost B.M., Flemming I. (1991). Synthesis of quinones. Comprehensive Organic Synthesis.

[13] Gallagher P.T. (1996). The synthesis of quinones. Contemp. Org. Synth..

[14] Akai S., Kita Y. (1998). Review of recent progress in the synthesis of *p*-quinones and *p*-dihydroquinones through oxidation of phenol derivatives. Org. Prep. Proced. Int..

[15] Deya P.M., Dopico M., Raso A.G., Morey J., Saa J.M. (1987). On the regioselectivity of the Fremy’s salt oxidation of phenols. Tetrahedron.

[16] Saladino R., Neri V., Mincione E., Marini S., Coletta M., Fiorucci C., Filippone P. (2000). A new and efficient synthesis of ortho- and para-benzoquinones of cardanol derivatives by the catalytic system MeReO_3_-H_2_O_2_. J. Chem. Soc. Perkin Trans. 1.

[17] Crandall J.K., Zucco M., Kirsch R.S., Coppert D.M. (1991). The formation of orthoquinones in the dimethyldioxirane oxidation of phenols. Tetrahedron Lett..

[18] Barton D.H.R., Finet J.P., Thomas M. (1988). Comparative oxidation of phenols with benzeneseleninic anhydride and with benzeneseleninic acid. Tetrahedron.

[19] Magdziak D., Rodriguez A.A., van de Water R.W., Pettus T.R.R. (2002). Regioselective oxidation of phenols to *o*-quinones with *o*-iodoxybenzoic acid (IBX). Org. Lett..

[20] Pezzella A., Lista L., Napolitano A., d’Ischia M. (2005). An expedient one-pot entry to catecholestrogens and other catechol compounds via IBX-mediated phenolic oxygenation. Tetrahedron Lett..

[21] Bernini R., Crisante F., Barontini M., Fabrizi G. (2009). A new and efficient route for the synthesis of naturally occurring catecholamines. Synthesis.

[22] Bernini R., Mincione E., Crisante F., Barontini M., Fabrizi G. (2009). A novel use of the recyclable polymer-supported IBX: An efficient chemoselective and regioselective oxidation of phenolic compounds. The case of hydroxytyrosol derivatives. Tetrahedron Lett..

[23] Barontini M., Bernini R., Crisante F., Fabrizi G. (2010). Selective and efficient oxidative modifications of flavonoids with 2-iodoxybenzoic acid (IBX). Tetrahedron.

[24] Wu A., Duan Y., Xu D., Penning T.M., Harvey R.G. (2010). Regiospecific oxidation of polycyclic aromatic phenols to quinones by hypervalent iodine reagents. Tetrahedron.

[25] Richardson R.D., Wirth T. (2006). Hypervalent iodine goes catalytic. Angew. Chem. Int. Ed. Engl..

[26] Zhdankin V.V., Stang P.J. (2008). Chemistry of polyvalent iodine. Chem. Rev..

[27] Ochiai M., Miyamoto K. (2008). Catalytic version of and reuse in hypervalentorgano-λ^3^- and -λ^5^-iodane oxidation. Eur. J. Org. Chem..

[28] Dohi T., Kita Y. (2009). Hypervalent iodine reagents as a new entrance to organocatalysts. Chem. Commun..

[29] Uyanik M., Ishihara K. (2012). Catalysis with insitu-generated (hypo)iodite ions for oxidative coupling reactions. ChemCatChem.

[30] Yakura T., Konishi T. (2007). A novel catalytic hypervalent iodine oxidation of *p*-alkoxyphenols to *p*-quinones using 4-iodophenoxyacetic acid and Oxone^®^. Synlett.

[31] Yakura T., Yamauchi Y., Tian Y., Omoto M. (2008). Catalytic hypervalent iodine oxidation of *p*-dialkoxybenzenes to *p*-quinones using 4-iodophenoxyacetic acid and Oxone^®^. Chem. Pharm. Bull..

[32] Yakura T., Tian Y., Yamauchi Y., Omoto M., Konishi T. (2009). Catalytic hypervalent iodine oxidation using 4-iodophenoxyacetic acid and Oxone^®^: Oxidation of *p*-alkoxyphenols to *p*-benzoquinones. Chem. Pharm. Bull..

[33] Uyanik M., Akakura M., Ishihara K. (2009). 2-Iodoxybenzenesulfonic acid as an extremely active catalyst for the selective oxidation of alcohols to aldehydes, ketones, carboxylic acids, and enones with Oxone^®^. J. Am. Chem. Soc..

[34] Uyanik M., Ishihara K. (2009). Hypervalent iodine-mediated oxidation of alcohols. Chem. Commun..

[35] Uyanik M., Ishihara K. (2010). 2-Iodoxybenzenesulfonic acid (IBS) catalyzed oxidation of alcohols. Aldrichim. Acta.

[36] Uyanik M., Ishihara K. (2012). 2-Iodoxy-5-methylbenzenesulfonic acid-catalyzed selective oxidation of 4-bromobenzyl alcohol to 4-bromobenzaldehyde or 4-bromobenzoic acid with Oxone^®^. Org. Syn..

[37] Uyanik M., Fukatsu R., Ishihara K. (2009). IBS-catalyzed oxidative rearrangement of tertiary allylic alcohols to enones with Oxone^®^. Org. Lett..

[38] Cui L.C., Liu K., Zhang C. (2011). Effective oxidation of benzylic and alkane C-H bonds catalyzed by sodium *o*-iodobenzenesulfonate with Oxone^®^ as a terminal oxidant under phase-transfer conditions. Org. Biomol. Chem..

[39] Tanaka Y., Ishihara T., Konno T. (2012). A new entry for the oxidation of fluoroalkyl-substituted methanol derivatives: Scope and limitation of the organoiodine(V) reagent-catalyzed oxidation. J. Fluor. Chem..

[40] Giurg M., Syper L., Mlochowski J. (2004). Hydrogen peroxide oxidation of naphthalene derivatives catalyzed by poly(bis-1,2-diphenylene) diselenide. Pol. J. Chem..

[41] Carreño M.C., Gonzáles-López M., Urbano A. (2006). Oxidative de-aromatization of *para*-alkyl phenols into *para*-peroxyquinols and *para*-quinols mediated by Oxone^®^ as a source of singlet oxygen. Angew. Chem. Int. Ed. Engl..

[42] Ojha L.R., Kudugunti S., Maddukuri P.P., Kommareddy A., Gunna M.R., Dokuparthi P., Gottam H.B., Botha K.K., Parapati D.R., Vinod T.K. (2009). Benzylic carbon oxidation by an *in situ* formed *o*-iodoxybenzoic acid (IBX) derivative. Synlett.

[43] Ding Z., Xue S., Wulff W.D. (2011). A succinct synthesis of the vaulted biarylkigandvanol via a dienone-phenol rearrangement. Chem. Asian J..

[44] Ludwik S. (1989). The Baeyer-Villiger oxidation of aromatic aldehydes and ketones with hydrogen peroxide catalyzed by selenium compounds. A convenient method for the preparation of phenols. Synthesis.

[45] Bell K.H., McCaffery L.F. (1993). Regioselectivemonomethylation of unsymmetrical naphthalenediols with methanolic hydrogen chloride. Aust. J. Chem..

[46] Crich D., Zou Y. (2005). Catalytic oxidation adjacent to carbonyl groups and at benzylic positions with a fluorousseleninic acid in the presence of iodoxybenzene. J. Org. Chem..

[47] Suchard O., Kane R., Roe B.J., Zimmermann E., Jung C., Waske P.A., Mattay J., Oelgemöller M. (2006). Photooxygenations of 1-naphthols: An environmentally friendly access to 1,4-naphthoquinones. Tetrahedron.

[48] Perumal P.T., Bhatt M.V. (1980). Oxidation of halophenols and highly substituted phenols with lead(IV) acetate. Synthesis.

[49] Ogata T., Okamoto I., Kotani E., Takeya T. (2004). Biomimetic synthesis of the dinaphthofuranquinone violet-quinone, utilizing oxidative dimerization with the ZrO_2_/O_2_ system. Tetrahedron.

[50] Bernd P., Meike N., Anja P. (2004). The acid accelerated ruthenium-catalyzed dihydroxylation. Scope and limitations. Org. Biomol. Chem..

[51] Ratnikov M.O., Farkas L.E., McLaughlin E.C., Chiou G., Choi H., El-Khalafy S.H., Doyle M.P. (2011). Dirhodium-catalyzed phenol and aniline oxidations with T-HYDRO. Substrate scope and mechanism of oxidation. J. Org. Chem..

[52] Miyamura H., Shiramizu M., Matsubara R., Kobayashi S. (2008). Aerobic Oxidation of Hydroquinone Derivatives Catalyzed by Polymer-Incarcerated Platinum Catalyst. Angew. Chem. Int. Ed. Engl..

[53] Lebrasseur N., Fan G.J., Oxoby M., Looney M.A., Quideau S. (2005). λ^3^-Iodane-mediated arenol dearomatization. Synthesis of five-membered ring-containing analogues of the aquayamycin ABC tricyclic unit and novel access to the apoptosis inducer menadione. Tetrahedron.

